# A Method of Examining the Pulmonary Apex

**Published:** 1903-07

**Authors:** 


### A Method of Examining the Pulmonary Apex.

Auld has devised a method of examining the pulmonary apex, which he thinks yields trustworthy results in the very earliest stage of phthisis. The apices of the lungs extend on an average from 1 inch to 1½ inches above the clavicles and in some cases 2 inches, the right apex being a shade higher than the left. The method consists in outlining the resonant area above the clavicles. The key to the detection of incipient diseases in the apex is familiarity with the physical signs yielded in health. With deep inspiration, the upper area of resonance is a quarter of an inch higher than during forcible expiration. If the pulmonary apices be examined in this comparative manner, any deviation from the normal standard, any evident disproportion, either in respect of the characters common to both sides, may be justly regarded as a ground for suspicion. The early indications of abnormalities are (1) failure of the upper line of resonance on one or the other side to ascend during a deep inspiration; (2) a lowering of the upper line of resonance in whole or in part as compared with that of the opposite side; and (3) indistinct definition of the upper or outer line of resonance.—*London Lancet.*

